# Method for Isolating Hypericin from *Hypericum perforatum* and Preparing Its Micelles for Biomedical Applications

**DOI:** 10.3390/molecules31122048

**Published:** 2026-06-11

**Authors:** Dmitry Medvedev, Vasilisa Dalinina, Polina Golik, Polina Lavrinova, Ekaterina Plotnikova, Maksim Usachev, Veronika Usatova, Mikhail Grin, Tatiana Abakumova, Petr Ostroverkhov

**Affiliations:** 1 Department of Chemistry and Technology of Biologically Active Compounds, Medicinal and Organic Chemistry, Institute of Fine Chemical Technologies, MIREA-Russian Technological University, 86 Vernadsky Avenue, 119571 Moscow, Russia; dy.medvedev@mail.ru (D.M.); jafk0@yandex.ru (P.G.); lavrinova.p@mail.ru (P.L.); plotnikovaekaterina62@gmail.com (E.P.);; 2 Frumkin Institute of Physical Chemistry and Electrochemistry of Russian Academy of Sciences, 119071 Moscow, Russia; 3 Laboratory of Synthetic Neurotechnologies, Pirogov Russian National Medical University, 117997 Moscow, Russia; dalinina_vd4@rsmu.ru (V.D.); abakumova_to@rsmu.ru (T.A.); 4 National Medical Research Radiological Centre of the Ministry of Health of the Russian Federation, P.A. Hertsen Moscow Oncology Research Institute, 125284 Moscow, Russia; 5 Federal Center of Brain Research and Neurotechnologies, Federal Medical Biological Agency, 117997 Moscow, Russia; usatova.v@fccps.ru

**Keywords:** hypericin, photodynamic therapy, photosensitizer, micelles, Pluronic F-127, glioblastoma

## Abstract

Hypericin (Hyp) is a naturally occurring photosensitizer (PS) exhibiting a broad spectrum of biological activities. However, its high hydrophobicity significantly limits its medical applicability. This study aimed to develop an efficient method for extracting Hyp from *Hypericum perforatum* L. biomass and to obtain its water-soluble micellar formulation. A protocol for Hyp isolation from the aerial parts of the plant was established, involving a preliminary defatting step using a Soxhlet apparatus, followed by ultrasonic-assisted extraction with acetone. The water-soluble formulation was prepared via the thin-film hydration method using the nonionic block copolymer Pluronic F-127. The resulting micelles demonstrated colloidal stability, with a mean hydrodynamic diameter of approximately 30 nm. A high Hyp loading efficiency was achieved, with an encapsulation efficiency (EE) of 95.4 ± 2.7%, yielding a concentration of 600.6 ± 16.6 µM within the micellar formulation. In vitro biological studies were performed using the murine GL261 glioblastoma cell line. The micellar Hyp formulation exhibited efficient time-dependent cellular accumulation and photoinduced cytotoxicity, with no observable dark toxicity. The proposed method enables the production of a stable Hyp formulation that retains its photosensitizing properties, thereby opening promising avenues for its application in photodynamic and sonodynamic therapies.

## 1. Introduction

Hyp is a natural red pigment belonging to the naphthodianthrone class. It is a component of the plant species *Hypericum perforatum*, commonly known as St. John’s wort, and is predominantly found in the black or dark red glands located in the flowers. The naphthodianthrone content in St. John’s wort varies depending on the growing conditions and the plant variety [1]. In addition to St. John’s wort, Hyp has also been detected in endophytic fungi and fungi of the genus *Dermocybe* [2].

One method of obtaining Hyp is through chemical synthesis. This is a multi-step process involving the preparation of emodin, its subsequent conversion into protohypericin, from which Hyp is formed via a photocatalytic reaction. Currently, numerous synthetic pathways for Hyp have been developed, some of which allow for a reasonably good yield of the product. However, further modification of these processes is still required to improve overall efficiency, reduce costs, and minimize environmental pollution [3,4].

An alternative to the synthetic method of obtaining Hyp is its isolation from natural sources via extraction. Methanol, ethanol, acetone, dichloromethane, petroleum ether, and various combinations of these solvents in different ratios are most commonly used as extractants. To enhance efficiency and increase the pigment yield, extraction can be carried out under heating, as well as with ultrasound or microwave treatment. The low cost and availability of plant material, along with the simple conditions of extraction, make this method highly attractive; however, it also has several disadvantages. Due to the extensive chemical composition of the genus *Hypericum* and the relatively low content of Hyp itself, the selective isolation and purification of the pigment in large quantities becomes problematic [5,6].

Hyp exhibits a broad spectrum of activity and has long been used in various fields of medicine. It has proven to be an effective antidepressant and has gained popularity for the treatment of mild to moderate depression [7]. Hyp is known to possess antibacterial and antimicrobial properties, making it applicable for combating infections [8]. Furthermore, it exhibits antiviral activity [9]. Hyp can also be considered a potential agent for the treatment of neurological disorders. It is capable of inhibiting the formation of amyloid beta plaques and interacting with τ-protein, thus holding promise as an agent against Alzheimer’s disease [10].

Hyp possesses photosensitizing properties and high photocytotoxicity; however, it is practically non-toxic in the dark. The pigment exhibits intense light absorption in the wavelength range of 400 to 600 nm, as well as a high quantum yield of singlet oxygen generation [11], making it an appropriate candidate for use as a PS in photodynamic therapy (PDT). Intracellular accumulation of Hyp due to its lipophilicity occurs predominantly in the cytoplasmic membranes of cell organelles such as the endoplasmic reticulum, mitochondria, lysosomes, and Golgi apparatus [12]. When Hyp is used as a PS, two pathways of tumor destruction induced by photodynamic therapy are possible: (1) direct killing of tumor cells through damage to cellular membrane systems or mitochondria, and (2) destruction of the tumor vascular system. The extent of Hyp's effect on the tumor strongly depends on the wavelength of light used to activate the PS and the availability of oxygen, from which reactive species are generated that trigger mechanisms of apoptosis and necrosis.

In addition to direct cytotoxicity, Hyp is capable of modulating the tumor microenvironment by altering cytokine profiles, which may enhance antitumor immunity and reduce metastasis [13,14]. PDT using Hyp as a photosensitizer demonstrates activity against such cancers as glioblastoma, head and neck cancer, bladder cancer, colorectal cancer, breast cancer, and leukemia [15].

The antitumor sensitizing effect of Hyp can also be activated using ultrasound waves of relatively low intensity, enabling the use of this pigment in sonodynamic therapy (SDT). SDT represents an alternative approach to PDT in cancer treatment. By employing ultrasound waves, which can penetrate deeply into tissues, researchers can overcome one of the major limitations of PDT: low light penetration depth into biological tissues. Several mechanisms of sonosensitization have been identified: the generation of reactive oxygen species (ROS), induction of apoptosis, enhancement of antitumor immunity, and cavitation. The initiation of apoptotic mechanisms under SDT conditions is associated with increased expression of FAS/FASL proteins, excessive accumulation of Ca^2+^ within mitochondria, as well as reduced expression of Bcl-2, which blocks the release of cytochrome c from mitochondria, and increased expression of Bax, which promotes this process. The enhancement of antitumor immunity is achieved through the conversion of M2 macrophages (anti-inflammatory) into M1 macrophages (pro-inflammatory), increased secretion of the cytokines TNF and IFN-γ, and accelerated maturation of dendritic cells in the tumor microenvironment [16].

One of the most important phenomena induced by ultrasound is acoustic cavitation, which is divided into two types: non-inertial cavitation (also referred to as stable cavitation) and inertial cavitation [17]. During stable cavitation, microbubbles formed in the liquid medium undergo oscillatory motion, generating flows in the surrounding fluid that can cause damage to cell membranes and alter the relative positioning of intracellular organelles. In the process of inertial cavitation, microbubbles collapse violently, leading to the release of a large amount of energy and the emission of light flashes, known as sonoluminescence [18]. The local temperature increase caused by the microbubble collapse promotes the decomposition of water molecules and the sensitizer, resulting in the formation of free radicals that initiate chain reactions and generate reactive oxygen species, which exert a cytotoxic effect on tumor cells [19]. Furthermore, the sonosensitizer can absorb sonoluminescence light, which further activates it via the PDT mechanism. This explains why Hyp can be used simultaneously as a photo- and sonosensitizer [20,21].

As previously mentioned, Hyp is a lipophilic molecule, which complicates its direct use in biological media and limits its applicability in cancer therapy. To overcome this drawback, water-soluble delivery systems are being developed. One such system is based on polyvinylpyrrolidone (PVP), characterized by high stability and therapeutic efficacy in in vivo studies [22,23].

Another approach to improving the water solubility of Hyp is its encapsulation into porous silica nanoparticles. This system is highly stable and allows for the gradual release of the compound; however, a significant amount of Hyp becomes irreversibly immobilized within the nanoparticles [24].

Low-density and high-density lipoproteins are also considered as delivery systems for Hyp. Low-density lipoproteins represent globular particles that are actively internalized by tumor cells. Nevertheless, faster intracellular release of Hyp from the particles and stronger photodynamic action have been demonstrated for delivery systems based on high-density lipoproteins [25].

Liposomes are highly appropriate as delivery systems due to their non-toxic biodegradable nature and their ability to achieve prolonged circulation in the bloodstream through PEG surface modification [26]. They penetrate tumor cells and can accumulate within them. To enable more effective delivery, liposomes responsive to external inducements are being developed [27]. For Hyp liposomal delivery systems, sensitive to temperature, enzyme activity, light, and changes in pH have been described [28,29].

Increased bioavailability of the hydrophobic compound can also be achieved using copolymers that are capable of forming micelles. Such delivery systems are highly effective, non-toxic, selective toward tumor cells, and enhance their sensitivity to the drug [30]. Micelles form via self-assembly upon reaching a certain polymer concentration in solution, which makes them convenient and simple to prepare [31].

In this work, we determined the optimal conditions for the isolation of Hyp from *Hypericum perforatum*, obtained micellar emulsions based on Pluronic F-127, and investigated the photophysical and biological properties of the pigments.

## 2. Results and Discussion

### 2.1. Isolation of Hyp from Hypericum perforatum L. Biomass

The relevance of developing preparative methods for the isolation of secondary metabolites from plant raw materials is primarily due to the complexity and economic impracticality of their complete chemical synthesis. The preparative amounts of Hyp obtained in this work from *Hypericum perforatum* L. biomass demonstrate the potential of using plant raw materials as a source of the biologically active naphthodianthrone [32].

Existing synthetic strategies are largely associated with a number of limitations. First of all, they are characterized by multi-step processes and, consequently, low overall yields of around 6–9%, which makes them economically inefficient. Additionally, many approaches require the use of hard-to-reach reagents or harsh reaction conditions, including toxic catalysts and prolonged electromagnetic irradiation, significantly hindering the scalability of these processes. Attempts to optimize semi-synthetic approaches based on emodin dimerization have led to improved yields in the final stages. Nevertheless, the overall synthetic strategy remains technologically complex, imposes high demands on equipment, and exhibits high sensitivity to reaction conditions. The absence of a developed biotechnological framework for the metabolic synthesis of Hyp in plants also hinders the creation of microbiological systems for its biosynthesis [33].

**Scheme 1:**
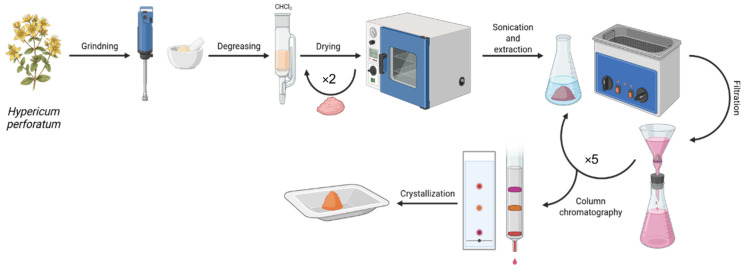
Isolation and purification of Hyp from *Hypericum perforatum* biomass.

A key feature of the scheme proposed in this work is the inclusion of a preliminary defatting step of the raw material with CHCl_3_ in a Soxhlet apparatus (Scheme 1). Although this step increases the overall isolation time, its use is justified when working with large biomass loads. The removal of chlorophylls, waxes, triglycerides, and a number of other lipophilic compounds at the initial stage substantially simplifies the subsequent chromatographic separation. Monitoring the completeness of defatting by the decolorization of the solvent is straightforward and does not require additional instrumental support at the preparatory stage, thereby enhancing the accessibility of the method for both laboratory and industrial applications.

The use of ultrasonic extraction for isolating Hyp from defatted biomass addresses several practical challenges simultaneously. First, ultrasonic cavitation effectively disrupts cell walls and releases compounds without additional heating, which is critically important when working with thermolabile bioactive substances. Second, performing cyclic extraction with solvent replacement every 30 min ensures complete isolation and does not require complex equipment. Acetone ((CH_3_)_2_CO) was chosen as the extractant due to its ease of removal and compatibility with subsequent chromatographic purification.

**Figure 1:**
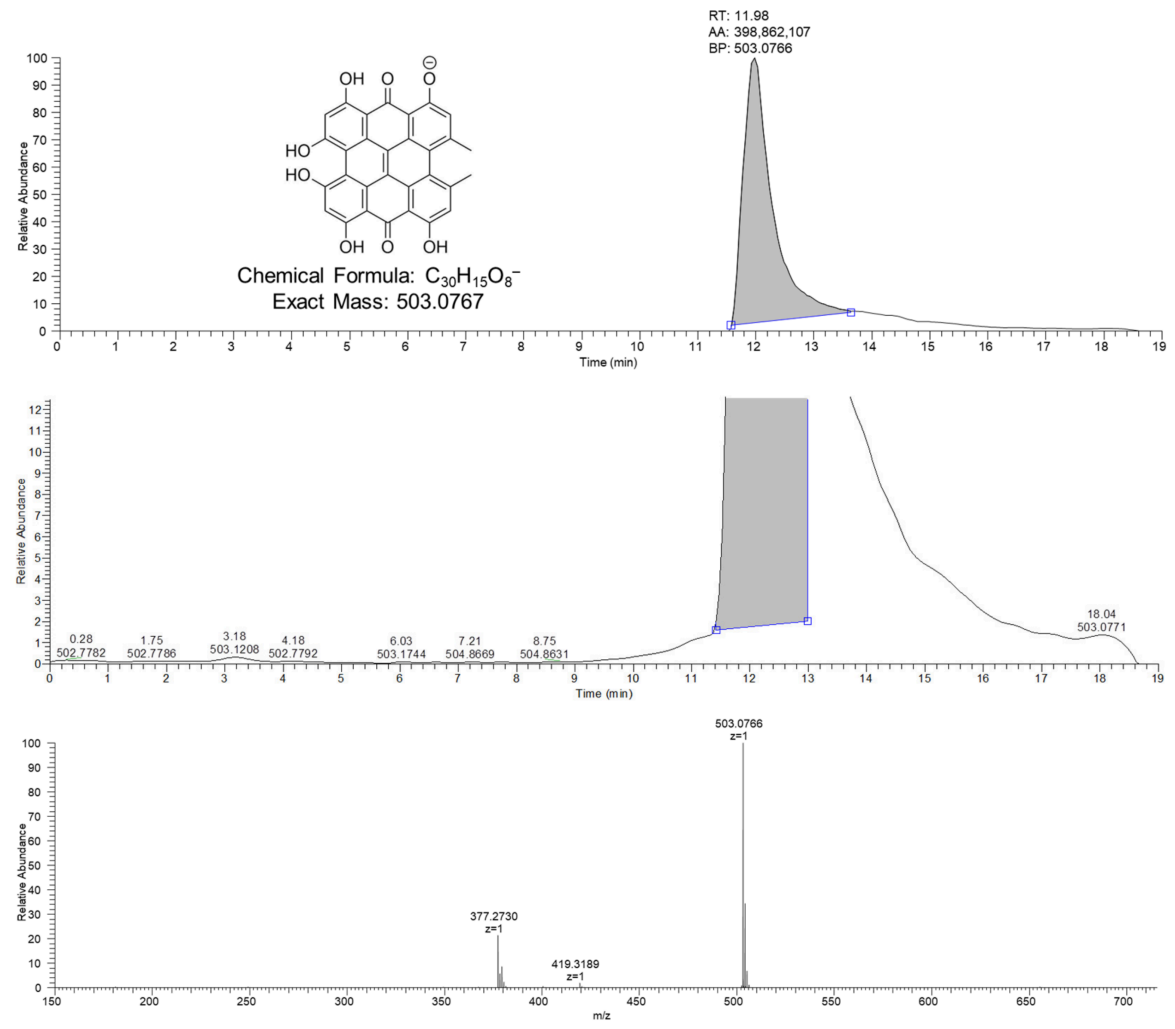
LC-HRMS chromatogram (base peak intensity) of Hyp. HESI ionization in negative electrospray mode. The chromatogram shows the complete baseline, with the detected ion for Hyp ([M-H]^−^ adduct) at *m*/*z* 503.0766 (experimental) and a retention time of 11.98 min. The calculated *m*/*z* for the deprotonated molecular ion [M-H]^−^ of Hyp (C_30_H_16_O_8_) is 503.0767, Δ = 0.6 ppm.

Identification of the isolated Hyp was performed using high-resolution chromatography-mass spectrometry. The analysis was carried out in negative ion acquisition mode, which is optimal for phenolic-type compounds. On the chromatogram, a peak corresponding to the deprotonated molecular ion [M-H]^−^ of Hyp is observed in the mass spectrum with an *m*/*z* ratio of 503.0719–503.0819. The experimentally determined mass corresponds to the calculated molecular formula of the analyzed compound, C_30_H_16_O_8_. Figure 1 shows the entire mass chromatogram from 0 to 19 min. The total analysis time, according to the given gradient separation program, taking into account the balancing of the column to the initial conditions, is 21 min. No signal was recorded during the equilibration of the column from 19 to 21 min. The structure of Hyp was also investigated using ^1^H and ^13^C NMR spectroscopy (Figures S1–S3).

**Figure 2:**
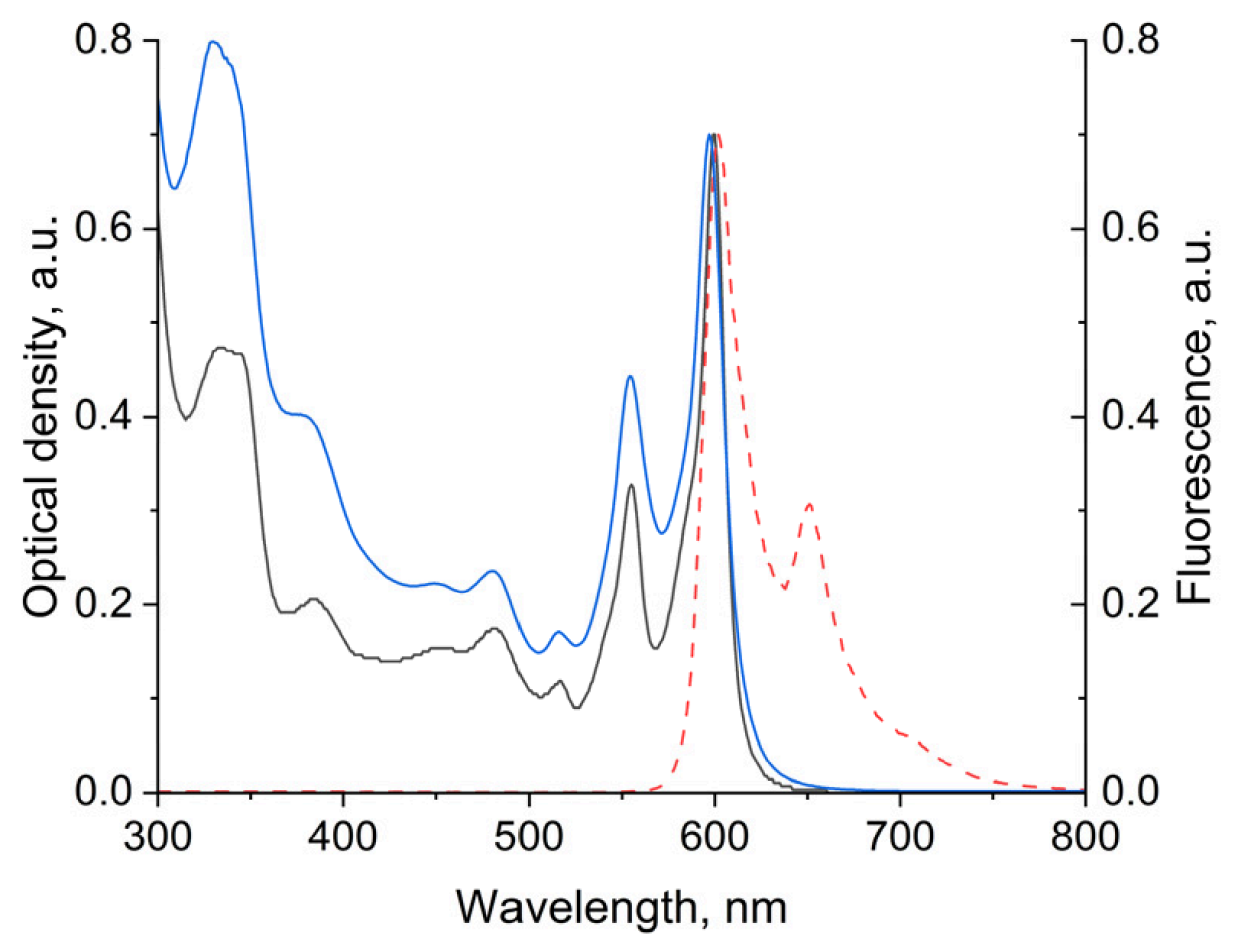
Normalized absorption (black solid line) and fluorescence (red dotted line, λ_exc_ = 350 nm) spectra of Hyp in acetone (black solid line) and in the micellar phase (blue solid line).

Since Hyp is a natural polycyclic quinone with intense fluorescence and photosensitizing activity, a key step in its physicochemical characterization was the investigation of its optical properties. To this end, the fluorescence and absorption spectra of the isolated compound were recorded (Figure 2).

In the absorption spectrum of Hyp, two characteristic bands are observed in the long-wavelength region with maxima at 555 and 600 nm, the latter corresponding to the quantum-allowed S_0_ → S_1_ transition and exhibiting the highest intensity, which is consistent with literature data [34]. The Stokes shift determined from the fluorescence spectrum is 2 nm, indicating minimal molecular reorganization in the excited state and a high rigidity of the chromophore system, characteristic of polycyclic aromatic compounds with an extensive π-system.

The yield of Hyp experimentally obtained in this work is consistent with literature data on the content of naphthodianthrones in the aerial parts of *Hypericum perforatum* L., which ranges from 0.05 to 0.3% calculated on a dry biomass basis [35]. The lower content relative to the upper limit of this range may be attributed to the geographic and climatic characteristics of the collection site of the raw material. Thus, the optimized extraction and chromatographic purification procedures for biomass described in this work allow Hyp to be obtained with reproducible characteristics, avoiding the complexities inherent in multi-step organic synthesis.

### 2.2. Preparation and Physicochemical Properties of Micellar Emulsions of Hyp with Pluronic F-127

It is known that the incorporation of drugs into Pluronic-based micelles increases their solubility and stability. Pluronics are symmetric polymers, triblock copolymers of ethylene oxide (polar part) and propylene oxide (nonpolar part), also known as proxanols and poloxamers. These cosolvents are pharmaceutical excipients registered in the United States and British Pharmacopoeias [36]. In particular, micelles based on Pluronic F-127 are widely used for the solubilization of hydrophobic anticancer drugs [37].

**Scheme 2:**
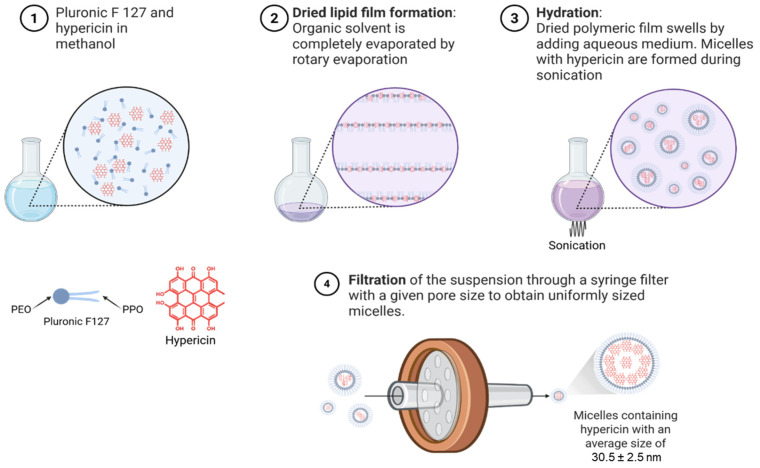
Preparation of micellar emulsions of Hyp with Pluronic F-127.

In this regard, Pluronic F-127 was used to prepare micellar emulsions containing Hyp. To obtain micelles and simultaneously load Hyp, a standard thin-film hydration method, commonly employed for the encapsulation of hydrophobic molecules, was applied, followed by extrusion through a 0.22 μm syringe filter (Scheme 2).

**Figure 3:**
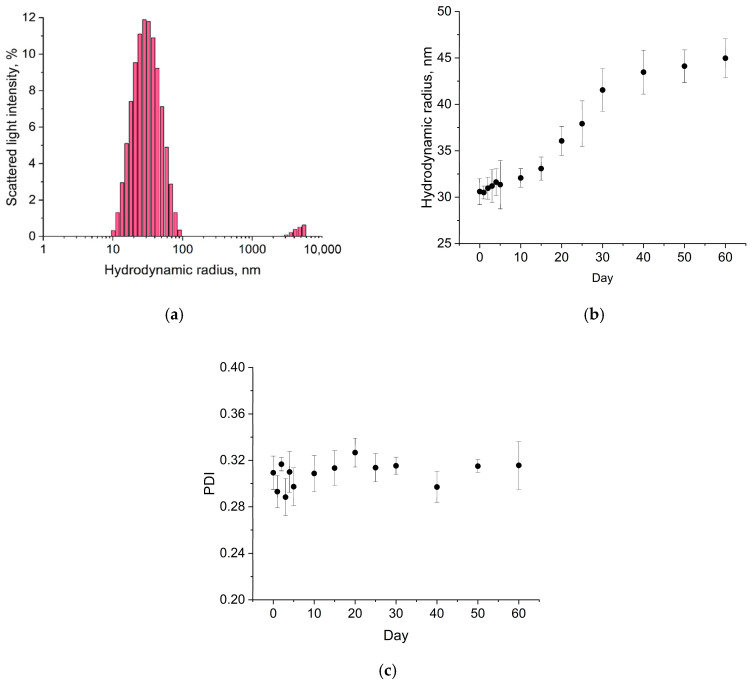
Sizes (**a**) and stability (**b**,**c**) of Hyp micelles.

The average hydrodynamic diameter of the micelles immediately after preparation was 30.5 ± 2.5 nm (Figure 3a). An important parameter to assess prior to biomedical application of micellar emulsions is stability against aggregation. The obtained micellar systems based on Pluronic F-127 with Hyp demonstrated stability when stored in the dark at a temperature not exceeding +4 °C for 60 days (Figure 3b,c).

During long-term storage, an increase in the hydrodynamic radius of the micelles was observed; however, this phenomenon is not accompanied by significant aggregation or sedimentation instability, nor does it lead to an increase in the polydispersity index (PDI), confirming that the colloidal stability of the system is maintained.

The quantitative content of Hyp in the micelles was determined spectrophotometrically. It was established that Hyp loading was quantitative in the system containing the nonionic solubilizer Pluronic F-127 at a mass concentration of 4%, corresponding to 600.6 ± 16.6 µM (α = 0.05, *n* = 5). The EE was determined to be 95.4 ± 2.7% (α = 0.05, *n* = 5). The retention of the spectral properties of Hyp after immobilization in micelles was further confirmed by fluorometry (Figure 2). In micellar solutions, an intense fluorescence in the range 600–650 nm was registered upon excitation at 350 nm. A distinctive feature compared with the free form of Hyp in acetone is a hyperchromic effect in the short-wavelength region of the absorption spectrum of the micellar solutions. This phenomenon may be attributed to changes in the polarity of the microenvironment and the formation of various types of aggregates within the hydrophobic core of the micelle, which is characteristic of aromatic molecular systems.

Thus, it can be concluded that the chosen method of Hyp solubilization meets the requirements for systems intended for biomedical applications. Accordingly, the obtained micellar systems containing Hyp were used for in vitro biological studies.

### 2.3. In Vitro Studies of Hyp Micellar Emulsions

**Figure 4:**
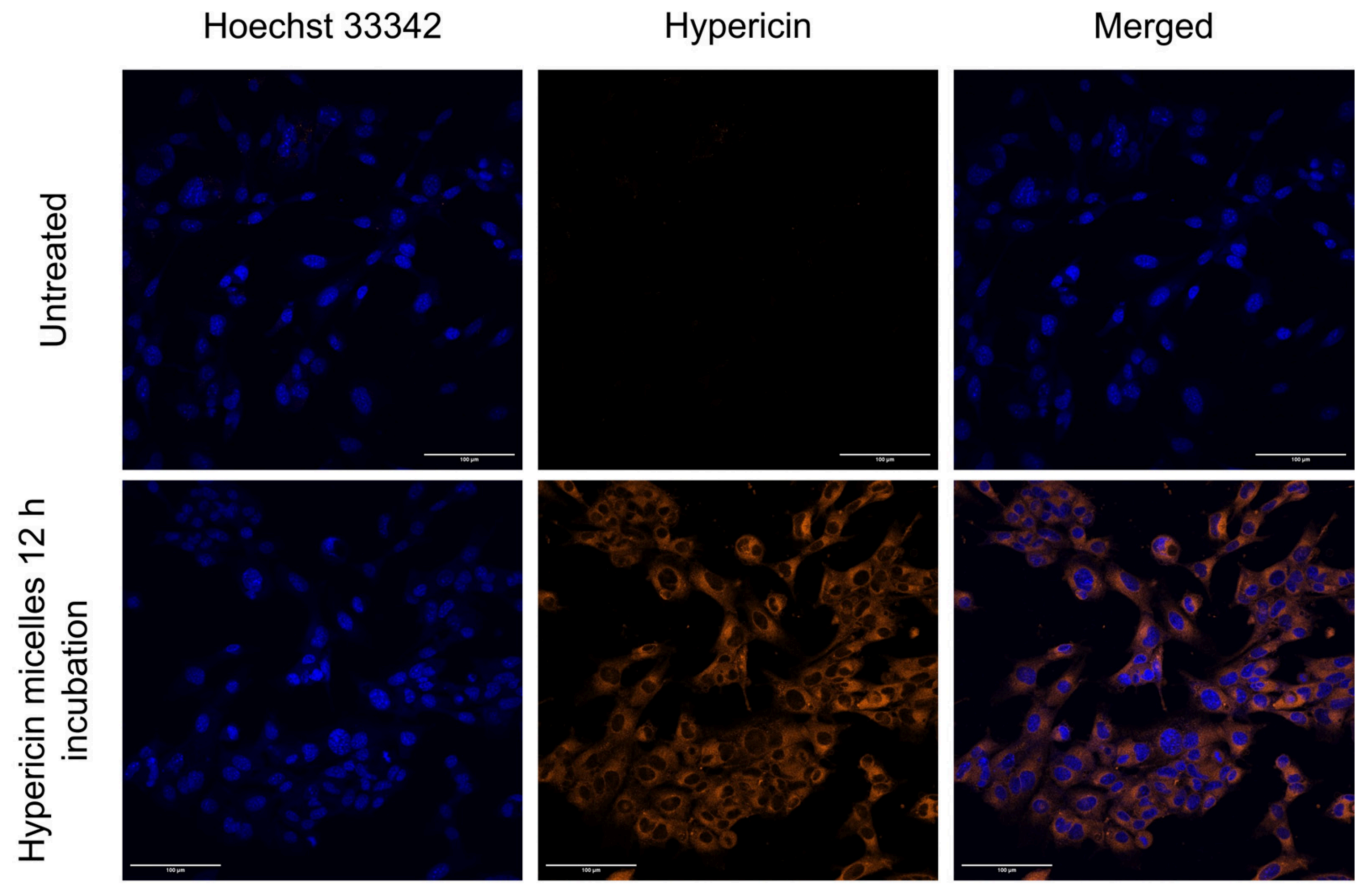
Analysis of intracellular accumulation of micellar Hyp in mouse glioblastoma GL261 cells with Hoechst 33342 nuclear staining (the blue color corresponds to the nuclei (DAPI), and the red color corresponds to the localization of Hyp).

It was demonstrated by using confocal fluorescence microscopy that the micellar form of Hyp effectively accumulates in mouse GL261 glioblastoma cells and exhibits cytoplasmic distribution (Figure 4).

Obtained micelles based on Pluronic F-127 have a small size, which enables their preferential accumulation in tumors through passive targeting, particularly due to the enhanced permeability and retention effect [38]. The hydrophilic shell consisting of polyethylene oxide chains in the poloxamer prevents rapid clearance of the particles by the mononuclear phagocyte system, while the micelle core composed of polypropylene oxide increases the bioavailability and cellular uptake of the hydrophobic compound [39]. Furthermore, some authors report that Pluronic F-127 can act as an inhibitor of ABC transporters (ATP-binding transport proteins), which hinder drug accumulation in tumor cells [40]. These factors account for the efficient uptake of micelles by GL261 cells and the high intracellular accumulation of Hyp, which serves as an important prerequisite for targeted photodynamic action.

In vitro studies demonstrated that the micellar form of Hyp induces concentration-dependent photoinduced death of GL261 glioblastoma tumor cells within the micromolar range (Table 1). With increasing incubation time, more effective inhibition of GL261 cell proliferation was observed; the maximum photoactivity was achieved after a 12 h incubation, with an IC_50_ of 0.97 ± 0.08 μM. Increasing the incubation time to 24 h did not lead to a statistically significant decrease in the IC_50_ value. It should be noted that the photoinduced activity was mediated solely by intracellularly accumulated Hyp, since the medium containing it was replaced with fresh medium prior to irradiation.

In vitro studies on DF-2 dermal fibroblasts showed that the micellar form of Hyp exhibits lower photoinduced activity (Table 1): the IC_50_ value after 24 h of incubation with the cells was 17.58 ± 0.74 μM, which is 20.4 times higher than that for GL-261 tumor cells.

Using the MTT and resazurin assays, it was found that in the absence of light exposure, the micellar form of Hyp did not exhibit cytostatic or cytotoxic effects on mouse GL261 glioblastoma cells and human dermal fibroblasts DF-2 within the investigated concentration range.

Thus, in the absence of light, Hyp exhibits minimal or no toxicity. However, upon irradiation, intracellular Hyp primarily activates type I and II PDT pathways, triggering the formation of singlet oxygen and other ROS. This localized generation of reactive species induces severe oxidative stress, leading to irreversible damage to cell membranes and organelles [41], ultimately causing pronounced photoinduced cytotoxicity against GL261 glioma cells.

## 3. Materials and Methods

### 3.1. Solvents and Equipment

The following solvents were used: acetone ((CH_3_)_2_CO), dichloromethane (CH_2_Cl_2_), chloroform (CHCl_3_), hexane, methanol (CH_3_OH), and dimethyl sulfoxide (DMSO) (Khimmed, Moscow, Russia). The solvents were purified and prepared according to standard procedures. HPLC-grade acetonitrile (Merck KGaA, Darmstadt, Germany) was used for chromatography-mass spectrometric analyses.

The following equipment was used: Sartorius analytical electronic balance with a weighing accuracy of 0.0001 g (Sartorius AG, Niedersachsen, Göttingen, Germany); VLA-MA analytical semi-micro balance with a weighing accuracy of 0.00001 g (NPP Gosmetr LLC, St. Petersburg, Russia); rotary evaporator (IKA-Werke GmbH & Co. KG, Baden-Württemberg, Staufen, Germany); Elmasonic S100H ultrasonic bath (Elma, Baden-Württemberg, Singen, Germany); Stegler LM-250 laboratory mill (Stegler, Guangzhou, China); ALUGRAM Xtra SIL G/UV254 plates for analytical thin-layer chromatography (TLC) coated with silica gel 60 (0.2 mm) (Macherey-Nagel, Nordrhein-Westfalen, Düren, Germany). Preparative chromatography was performed both by column chromatography on Silica gel 60 (0.0040–0.0063 mm) (Merck KGaA, Hessen, Darmstadt, Germany) and using glass-backed chromatographic plates (20 × 20 cm) coated with the same silica gel.

Absorption and fluorescence spectra were recorded using a UV1800 UV/VIS spectrophotometer (Shimadzu, Duisburg, Germany) and an RF-5301 spectrofluorometer (Shimadzu, Duisburg, Germany) in quartz cuvettes (1.0 × 1.0 cm) with an optical path length of 1 cm (spectral slit width 1 nm) at 25 °C. Background absorption of the corresponding solvents was subtracted automatically.

Chromatographic separation was performed on a Dionex UltiMate RS 3000 UHPLC system (Thermo Scientific, Dreieich, Germany). The system was equipped with a binary pump, a solvent degasser, an autosampler, and a column thermostat. Separation was achieved using a Nucleodur C-18 column (100 mm × 2.0 mm, 1.8 μm particle size; Macherey-Nagel, Düren, Germany) maintained at 40 °C. The mobile phase consisted of (A) 0.1% formic acid in water and (B) 0.1% formic acid in isopropanol. The following gradient was applied at a flow rate of 0.4 mL/min: 0–2 min, 100% B; 2–15 min, linear gradient from 100% to 5% B; 15–18 min, 5% B; 18–18.01 min, return to 100% B; 18.01–21 min, 100% B for column re-equilibration. The autosampler temperature was set to 4 °C, and the injection volume was 1.0 μL.

Mass spectrometric detection was performed using a Q-Exactive HF-X hybrid quadrupole-Orbitrap mass spectrometer (Thermo Scientific, Darmstadt, Germany) equipped with a heated electrospray ionization source (HESI-II). The instrument was operated in both positive and negative ionization modes with a resolution of 30,000 (at *m*/*z* 200). The scanning range was *m*/*z* 250–2000. Source parameters: spray voltage in positive mode 4.1 kV, negative mode −3.5 kV; transfer capillary temperature = 350 °C; sheath gas flow rate = 45 arb. units; auxiliary gas flow rate = 25 arb. Units. Data-dependent MS^2^ acquisition was enabled for the top 5 most intense ions using a normalized collision energy (NCE) of 20, 25, and 30%. Data acquisition and processing were performed using Xcalibur software (version 4.7, Thermo Scientific).

Micellar emulsions were investigated and characterized by dynamic light scattering (DLS). Measurements were carried out on a Zetasizer Nano ZS analyzer (Malvern, Worcestershire, UK) at 25 °C. The obtained data were processed using Zetasizer software (version 8.02).

### 3.2. Plant Material

Aerial parts of *Hypericum perforatum* L. (St. John’s wort) were collected during the full-flowering stage from July to August 2025 in forest areas of the southeastern Moscow region, which belong to the category of protective forests of green zones and water protection zones of the Oka and Osetr rivers, at locations remote from sources of anthropogenic pollution, following the general rules for the collection of medicinal plants. Branches with inflorescences were dried at a temperature of 25 ± 3 °C under constant ventilation until constant mass was achieved. The obtained biomass was ground using a laboratory mill to a homogeneous powder with a particle size of less than 1 mm and stored in sealed, light-protected containers at 20–25 °C until extraction.

### 3.3. Preparative Extraction of Lipophilic Impurities

To simplify subsequent chromatographic purification, the preliminary removal of lipophilic compounds (chlorophylls, waxes, triglycerides) and partial disruption of cell walls were carried out using continuous extraction in a Soxhlet apparatus. The prepared and ground biomass (30.0 g) was placed into a glass filtering crucible for solid-phase extraction, which was then installed in the extraction chamber of the Soxhlet apparatus. Chemically pure grade CHCl_3_ (600 mL) was used as the extractant. Extraction was performed over two consecutive cycles of 9 h each. The criterion for cycle completion was complete decolorization of the solvent in the extractor siphon. Between cycles, the biomass was removed and dried in a fume hood at 20–25 °C until constant mass was achieved. After the second cycle, the biomass was dried in a fume hood under the same temperature conditions for 12 h to remove trace amounts of the extractant.

### 3.4. Ultrasonic Extraction of Hyp

Hyp was extracted from the defatted biomass using acetone as a polar organic solvent. The residue dried after chloroform treatment was quantitatively transferred into a 500 mL conical flask, and 150 mL of acetone was added, corresponding to a raw material/extractant ratio of 1:4 (*w*/*w*). The process was carried out in an ultrasonic bath at a temperature of 25 ± 5 °C in the absence of light. Extraction was performed over 5 repeated cycles, each lasting 30 min. After each cycle, the suspension was filtered under vacuum through a paper filter, the solid residue was returned to the flask, and a fresh portion of acetone was added. The combined acetone fractions, which exhibited a characteristic dark red color indicating the presence of naphthodianthrones, were concentrated on a rotary evaporator.

### 3.5. Chromatographic Purification of Hyp

Purification of Hyp from accompanying compounds was carried out using normal-phase column chromatography. The concentrate of the acetone fractions was dissolved in a minimal amount (5–10 mL) of a (CH_3_)_2_CO/CH_3_OH mixture (1:1, *v*/*v*) and adsorbed onto 3.0 g of silica gel. The solvent was removed from the resulting suspension under reduced pressure. The dry mass was collected and loaded onto a prepared chromatographic column. Elution was performed in a gradient system of (CH_3_)_2_CO/CH_3_OH/CH_2_Cl_2_ ranging from a ratio of 1:3:10 to 1:2:20 (*v*/*v*/*v*). The process was monitored by TLC based on the characteristic dark red coloration of the Hyp fraction under visible light and intense fluorescence upon irradiation with UV light at a wavelength of 365 nm. As an additional identification method, spectrophotometric analysis of the eluate was performed, since Hyp exhibits characteristic long-wavelength absorption maxima at 550 and 590 nm. Fractions containing Hyp were combined and concentrated on a rotary evaporator. The residue was recrystallized from hexane on a watch glass to obtain dark-burgundy crystals. The yield of Hyp was 20.8 mg (0.069% relative to a dry biomass basis).

### 3.6. Preparation of Micellar Water-Soluble Forms of Hyp

A micellar solution of Hyp in water was obtained using the thin-film hydration method, employing the nonionic amphiphilic copolymer Pluronic F-127 (Merck, Sigma Aldrich, St. Louis, MO, USA) as a solubilizer. Accurately weighed portions of Hyp (0.6 mg) and the solubilizer (80.0 mg) were dissolved in a minimal amount of CH_3_OH (2.0 mL) and transferred into a 50 mL round-bottom flask. The organic solvent was removed from the mixture using a rotary evaporator at a temperature of 36.0 °C and a rotation speed of 110 rpm for 30 min until a uniform dry purple-tinted film formed on the flask walls. Subsequently, 1.919 g of 0.9% sodium chloride solution (Grotex LLC, St. Petersburg, Russia) was added to the flask, and the resulting film was subjected to ultrasonic hydration at 25 °C for 10 min. To complete the solubilization processes and system relaxation, the resulting colloidal solution was kept at room temperature in the dark for 1 h. To remove insoluble aggregates, the Hyp solution with a mass concentration of 0.3 mg/mL was filtered through a 0.22 μm Millipore membrane filter under aseptic conditions and stored in a cool, dark place at a temperature not exceeding +4 °C, protected from light.

The EE was determined after filtration through a 0.22 μm Millipore membrane filter using the formula:

$$EE=\frac{m_{Hyp}^{Micelles}}{m_{Hyp}^{\Sigma }}\cdot 100\%,$$

where $m_{Hyp}^{Micelles}$ is the mass (mg) of Hyp in the filtrate after passage through a 0.22 μm membrane filter, and $m_{Hyp}^{\Sigma }$ is the total mass (mg) of Hyp initially weighed for the preparation of the micellar formulation.

### 3.7. Stability Study of the Water-Soluble Form of Hyp

The size and stability of Hyp micellar emulsions were studied using a Zetasizer Nano ZS instrument (Malvern, Worcestershire, UK). All measurements were performed using a single protocol: temperature—25 °C, cuvette material—polystyrene, number of measurements—3, solvent—water. The data were averaged and subjected to statistical processing. To determine stability, the size and PDI of the emulsions were measured on days 1, 2, 3, 4, 5, 10, 15, 20, 25, 30, 40, 50, and 60 after preparation. Between measurements, the emulsions were stored at a temperature not exceeding 4 °C in the dark.

### 3.8. In Vitro Studies

#### 3.8.1. Characterization of In Vitro Test Systems

GL261 murine glioma cell lines were kindly provided by Dr. Aleksei Stepanenko from the Department of Fundamental and Applied Neurobiology of V. P. Serbsky Federal Medical Research Center of Psychiatry and Narcology. The DF-2 cell line (human dermal fibroblasts) was obtained from the Russian Collection of Typical Cell Cultures (Moscow, Russia). GL261 and DF-2 cells were cultured in DMEM (PanEco, Moscow, Russia) and DMEM/F12 (Capricorn Scientific, Ebsdorfergrund, Germany) medium, respectively, supplemented with L-glutamine (Capricorn Scientific, Ebsdorfergrund, Germany), 10% FBS (Biowest, Nuaillé, France), and 100 U/mL penicillin–streptomycin (PanEco, Moscow, Russia) under standard conditions at 37 °C in a humidified atmosphere containing 5% CO_2_. During passaging, cells were detached from the flask surface using a 0.25% trypsin solution (PanEco, Moscow, Russia).

#### 3.8.2. Evaluation of Hyp Accumulation in GL261 Mouse Glioblastoma Cells

GL261 cells were seeded into glass-bottom culture Petri dishes (d = 35 × 10 mm, SPL Life Sciences, Pocheon-si, Republic of Korea) at a density of 5 × 10^5^ cells in complete growth medium. After 24 h, the medium was replaced with a fresh one containing the micellar form of Hyp at a concentration of 10 μM. The cells were then incubated with Hyp for 12 h; afterwards, the culture medium was removed, and the cells were washed twice with Hanks’ solution (PanEco, Moscow, Russia) and stained with Hoechst 33342 (Thermo Fisher Scientific, Waltham, WA, USA) according to the manufacturer’s protocol. Cell images were obtained using a Nikon Eclipse Ti2 confocal microscope (Nikon, Tokyo, Japan). The obtained images were processed using Fiji software (version 2.16.0) [42].

#### 3.8.3. Study of Photoinduced and Cytotoxic Activity of Hyp

To study the photoinduced activity, GL261 and DF-2 cells were seeded into 96-well plates (NEST Biotechnology, Wuxi, China) at a density of 8 × 10^3^ and 10 × 10^3^ cells per well in 100 µL of complete growth medium, respectively, and treatment was performed 24 h after seeding. The micellar Hyp solution was added at final concentrations ranging from 0.15 to 40 µM with four replicates and three independent experiments. The incubation time of cells with Hyp prior to irradiation was 2, 4, 6, 12, and 24 h. Before irradiation, the medium containing Hyp was removed and replaced with fresh complete culture medium. Light exposure was performed using a diode lamp with a wavelength of 580 ± 20 nm (Moscow, Russia) at a power density of 20.0 mW/cm^2^, with a light dose of 10.0 J/cm^2^. After irradiation, the plates were placed in a CO_2_ incubator for 24 h. The photoinduced activity of Hyp was assessed after 24 h.

To evaluate the cytotoxic effect, cells were incubated with Hyp for 24 h in the dark in a CO_2_ incubator without irradiation at concentrations ranging from 0.01 to 100 µM. Control cells were not subjected to any treatment.

Cell viability was assessed using colorimetric MTT (thiazolyl blue tetrazolium bromide, Sisco Research Laboratories Pvt. Ltd., Mumbai, India) and resazurin (Alamar Blue, Yeasen, Gaithersburg, MD, USA) assays according to the manufacturers’ protocols.

A biologically significant effect was considered as inhibition of cell growth in culture by more than 50% (IC_50_).

IC_50_ values were calculated using GraphPad Prism 8 based on data from three independent experiments. Quantitative data are expressed as mean values ± confidence interval.

#### 3.8.4. Statistical Analysis

For all quantitative data, the standard deviation and confidence interval were calculated. Statistical analysis was performed using Statistica software, version 10.0. Statistical significance was assessed using the Mann–Whitney test at *p* ≤ 0.05.

## 4. Conclusions

Hyp is known to be usable as a photo- or sonosensitizer for PDT or SDT. However, its extremely low solubility in water severely limits any biomedical application. Various approaches to the solubilization of Hyp under physiological conditions exist. In the present work, we developed a modified method for the isolation of Hyp from *Hypericum perforatum* plant biomass. The use of a preliminary defatting step significantly facilitated the subsequent chromatographic purification of Hyp. We also employed ultrasonic treatment of the defatted biomass to increase the efficiency of Hyp extraction from the plant material. The identity of the isolated Hyp was confirmed by high-resolution chromatography-mass spectrometry. The photophysical properties of the isolated compound, namely its absorption and fluorescence spectra, fully correspond to literature data.

Another important objective of this work was to obtain a water-soluble form of Hyp for use in subsequent biological testing. We successfully applied a well-known method for preparing micellar emulsions based on Pluronic F-127 to solubilize Hyp in water. The developed method is characterized by technical simplicity and enables the preparation of micelles with a reproducible nanometric size (approximately 30 nm). This offers a distinct advantage over conventional liposomal systems, whose average diameter typically exceeds 100 nm [43]. High Hyp loading levels in the micelles were achieved, with their concentration in the emulsions varying in the micromolar range. For comparison, copolymers with shorter hydrophilic chains, such as Pluronic P-84, often exhibit lower solubilizing capacity toward hydrophobic polycyclic molecules [44]. Polyvinylpyrrolidone-based delivery systems also suffer from low loading capacity; however, specific formulation techniques exist to achieve a higher Hyp content within these nanoparticles [45]. The resulting Pluronic F-127 emulsions maintain long-term colloidal stability, rendering this formulation highly convenient for subsequent biological assays. They are less prone to aggregation and structural disruption compared to DPPC-based liposomal systems, while also providing more effective stabilization of Hyp [46].

Primary biological studies of the obtained composition demonstrated effective accumulation of Hyp in mouse GL261 glioblastoma cells. The obtained water-soluble form of Hyp also exhibited photoinduced activity against GL261 cells. Importantly, the excellent biological inertness of the vehicle itself serves as a significant asset of Pluronic F-127. While alternative copolymers with shorter hydrophilic segments (such as Pluronic P-123 or P-84) can cause standalone cytotoxicity owing to their elevated hydrophobicity, empty Pluronic F-127 micelles show no negative impact on cell viability in dark controls [47]. This also favorably distinguishes this system from both cationic liposomal carriers, which can exert toxic effects on cells [48], and PVP, whose administration is associated with storage disease and granuloma formation [49]. Therefore, it can be concluded that Hyp incorporated into Pluronic F-127 micelles retains both its photophysical properties and light-mediated sensitizing capability.

All the above-mentioned indicate the great potential of the developed water-soluble form of Hyp for use as a photo- and sonosensitizer in PDT and SDT.

## Figures and Tables

**Table 1:** Photoinduced Hyp activity depending on incubation time.

IC_50_, μM				
Incubation time before irradiation, h				
2	4	6	12	24
Mouse glioblastoma cell culture GL261				
16.14 ± 0.75	13.23 ± 0.85	7.18 ± 0.59	0.97 ± 0.08	0.86 ± 0.05
Human dermal fibroblasts DF-2				
34.26 ± 1.36	29.17 ± 1.06	25.28 ± 1.09	20.02 ± 1.12	17.58 ± 0.74

## Data Availability

The data presented in this study are available from the authors.

## Supplementary Materials

The following supporting information can be downloaded at: https://www.mdpi.com/article/10.3390/molecules31122048/s1, Figure S1: Numbering of atoms in the structure of Hyp; Figure S2: ^1^H NMR spectrum of Hyp in acetone-d_6_; Figure S3: ^13^C NMR spectrum of Hyp in acetone-d_6_.

## Abbreviations

The following abbreviations are used in this manuscript:

DLS	Dynamic Light Scattering
DMSO	Dimethyl Sulfoxide
Hyp	Hypericin
PDI	Polydispersity Index
PDT	Photodynamic Therapy
PS	Photosensitizer
PVP	Polyvinylpyrrolidone
ROS	Reactive Oxygen Species
SDT	Sonodynamic Therapy
TLC	Thin-layer Chromatography
