# Supplementary material for: Method for Isolating Hypericin from Hypericum perforatum and Preparing Its Micelles for Biomedical Applications
Source: Molecules. 2026 Jun 11;31(12):2048. doi: 10.3390/molecules31122048 (PMC13305305; doi:10.3390/molecules31122048)
Supplement: Supplementary file 1 [file molecules-31-02048-s001.zip › molecules-4293983-supplementary.pdf]

Supplementary materials

Method for Isolating Hypericin from *Hypericum perforatum* and Preparing Its Micelles for Biomedical Applications

Dmitry Medvedev [1,2], Vasilisa Dalinina [3], Polina Golik [1], Polina Lavrinova [1], Ekaterina Plotnikova [1,4], Maksim Usachev [1], Veronika Usatova [5], Mikhail Grin [1], Tatiana Abakumova [3,5] and Petr Ostroverkhov [1,*]

[1] Department of Chemistry and Technology of Biologically Active Compounds, Medicinal and Organic
Chemistry, Institute of Fine Chemical Technologies, MIREA-Russian Technological University, 86 Vernadsky Avenue, 119571 Moscow, Russia; dy.medvedev@mail.ru (D.M.); jafk0@yandex.ru (P.G.);
lavrinova.p@mail.ru (P.L.); plotnikovaekaterina62@gmail.com (E.P.); usachyov@mirea.ru (M.U.); grin@mirea.ru (M.G.)

[2] Frumkin Institute of Physical Chemistry and Electrochemistry of Russian Academy of Sciences, 119071 Moscow, Russia

[3] Laboratory of Synthetic Neurotechnologies, Pirogov Russian National Medical University, 117997 Moscow, Russia; dalinina_vd4@rsmu.ru (V.D.); abakumova_to@rsmu.ru (T.A.)

[4] National Medical Research Radiological Centre of the Ministry of Health of the Russian Federation, P.A. Hertsen Moscow Oncology Research Institute, 125284 Moscow, Russia

[5] Federal Center of Brain Research and Neurotechnologies, Federal Medical Biological Agency, 117997 Moscow, Russia; usatova.v@fccps.ru

* Correspondence: ostroverhov@mirea.ru

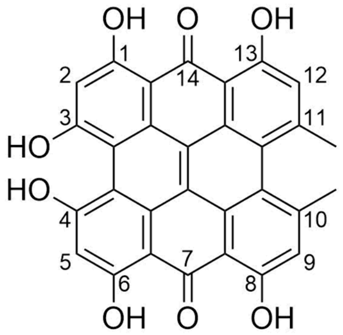

Figure S1 – Numbering of atoms in the structure of Hyp

^{1}H NMR (300 MHz, acetone-d$_6$, δ, ppm): 14.88 (H, s, 8-H), 14.82 (H, s, 13-H), 14.31 (H, s, 1-H), 14.23 (H, s, 6-H), 7.37 (2H, s, 9-H, 12-H), 6.63 (2H, s, 2-H, 5-H), 2.80 (6H, s, 10-CH$_3$, 11-CH$_3$).

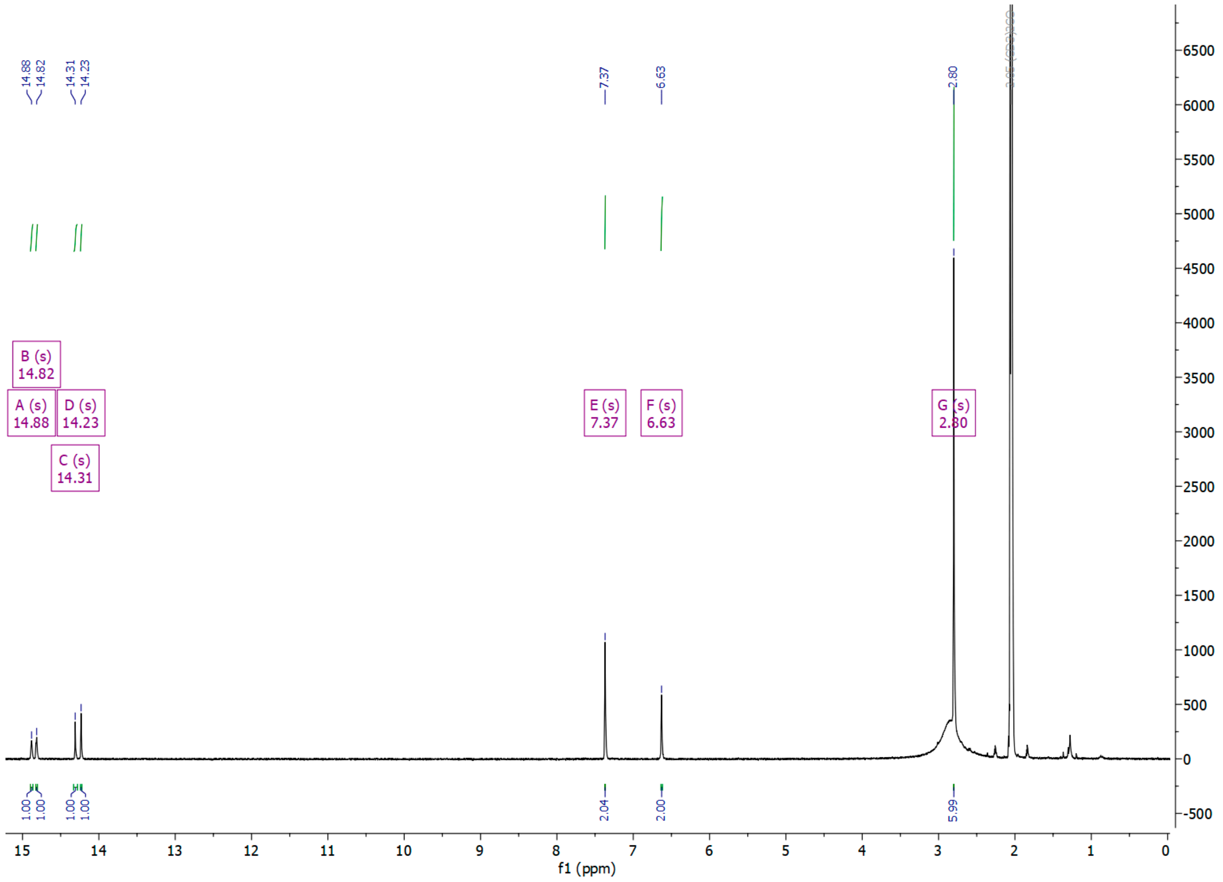

Figure S2 – ^{1}H NMR spectrum of Hyp in acetone-d$_6$

^{13}C NMR (300 MHz, acetone-d$_6$, δ, ppm): 185.26, 185.07, 176.64, 176.59, 170.10, 169.62, 163.00, 162.62, 144.24, 144.19, 128.61, 128.56, 127.87, 127.86, 123.00, 122.98, 122.14, 122.08, 121.21, 121.13, 119.52, 119.41, 110.07, 110.03, 106.62, 106.55, 103.35, 103.28, 24.02.

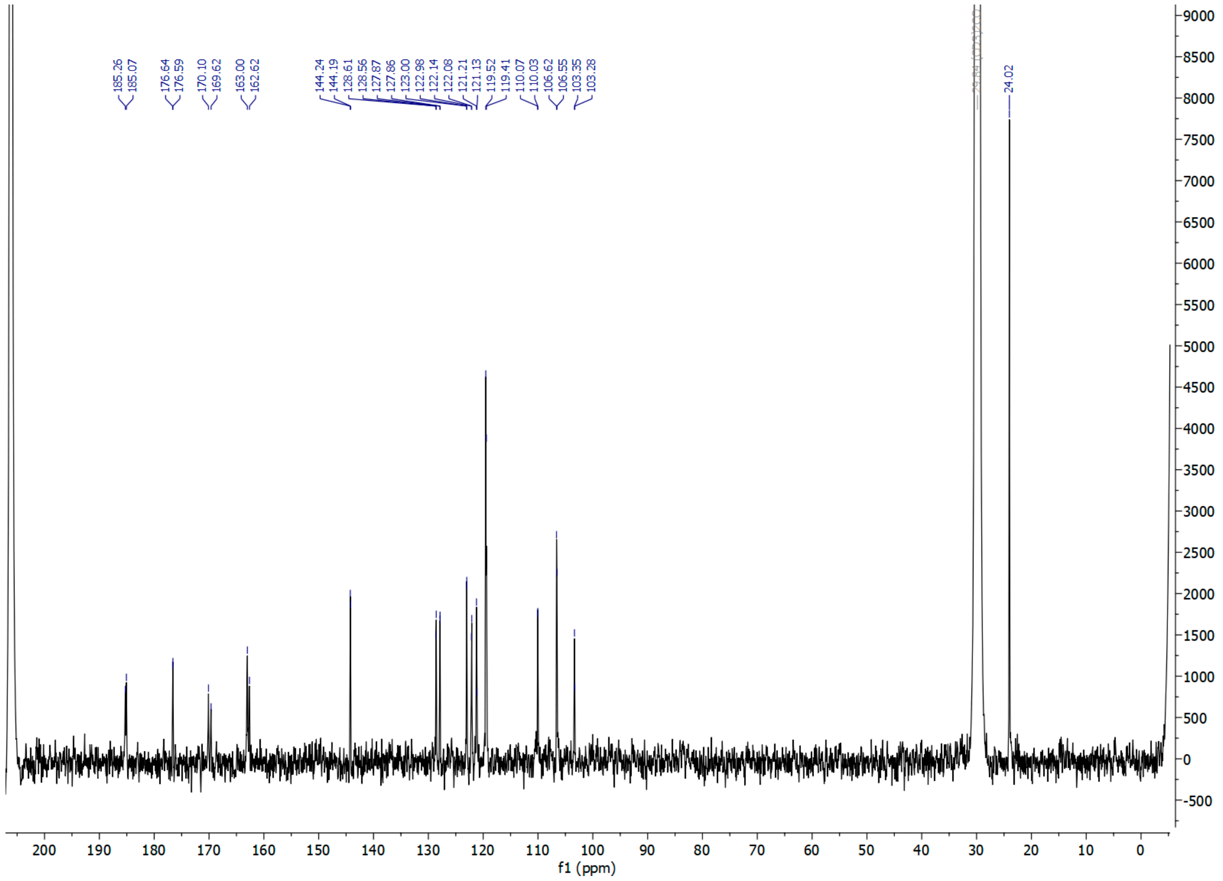

Figure S3 – ^{13}C NMR spectrum of Hyp in acetone-d_6
